# Prognostic value of hypoalbuminemia for adverse outcomes in patients with rheumatic heart disease undergoing valve replacement surgery

**DOI:** 10.1038/s41598-017-02185-2

**Published:** 2017-05-16

**Authors:** Xue-biao Wei, Lei Jiang, Yuan-hui Liu, Du Feng, Peng-cheng He, Ji-yan Chen, Dan-qing Yu, Ning Tan

**Affiliations:** 1 Department of Cardiology, Guangdong Cardiovascular Institute, Guangdong Provincial Key Laboratory of Coronary Heart Disease Prevention, Guangdong General Hospital, Guangdong Academy of Medical Sciences, Guangzhou, 510080 Guangdong China; 2 The Department of developmental biology, Harvard school of dental medicine, Harvard medical school, Boston, MA USA

## Abstract

High-risk patients with rheumatic heart disease (RHD) who were undergoing valve replacement surgery (VRS) were not identified entirely. This study included 1782 consecutive patients with RHD who were undergoing VRS to explore the relationship between hypoalbuminemia and adverse outcomes and to confirm whether hypoalbuminemia plays a role in risk evaluation. A total of 27.3% of the RHD patients had hypoalbuminemia. In-hospital deaths were significantly higher in the hypoalbuminemic group than in the non-hypoalbuminemic group (6.6% vs 3.1%, P = 0.001). Hypoalbuminemia was an independent predictor of in-hospital death (OR = 1.89, P = 0.014), even after adjusting for the Euro score. The addition of hypoalbuminemia to Euro score enhanced net reclassification improvement (0.346 for in-hospital death, P = 0.004; 0.306 for 1-year death, p = 0.005). A Kaplan-Meier curve analysis revealed that the cumulative rate of 1-year mortality after the operation was higher in patients with a new Euro score ≥6. These findings indicated that hypoalbuminemia was an independent risk factor for in-hospital and 1-year mortality after VRS in patients with RHD, which might have additive prognostic value to Euro score.

## Introduction

Rheumatic heart disease (RHD) is the permanent damage to the heart valves caused by an abnormal immune reaction to group A streptococcal infection. RHD is a global threat to human health, especially in developing countries^1^. Valve replacement surgery (VRS) is an effective therapy for RHD. Epidemiological data^2^ have indicated that 4.7% (698/14799) of patients died soon after cardiac surgery, among which 0.8% (36/4529) were low-risk patients (European System for Cardiac Operative Risk Evaluation, Euro score ≤2), 3.0% (182/5977) were medium-risk patients (Euro score 3–5) and 11.2% (480/4293) were high-risk patients (Euro score ≥6). The Euro score cannot, however, determine all high-risk patients^3^. Therefore, it is important to explore an index in which more high-risk patients can be identified.

Albumin is an important protein of human serum, and has multiple physiological functions in the body^4^. Hypoalbuminemia is common in patients with critical illnesses^5^. Hypoalbuminemia has been associated with poor prognosis in several conditions, such as acute coronary syndrome, acute aortic dissection, heart failure^6–8^, and in patients undergoing coronary artery bypass^9^. However, there are fewer reports that have investigated the relationship between low serum albumin (SA) and adverse outcomes in patients with RHD undergoing VRS. In addition, whether adding hypoalbuminemia to the Euro score can improve the prediction of poor outcomes is unclear. For these reasons, this study was conducted to assess the prognostic value of hypoalbuminemia in patients with RHD undergoing VRS.

## Methods

### Patient population

This was an observational, single-center study. Between March 2009 and July 2013, patients with RHD undergoing VRS were enrolled from Guangdong General Hospital, Guangzhou, China. All patients underwent coronary angiography to exclude coronary heart disease prior to the operation. Patients without SA level measured on admission was excluded in this analysis. Exclusion criteria also included if patients had any condition relating to hypoalbuminemia: (1) patients with end-stage renal disease (estimated glomerular filtration rate (eGFR) <30 mL/min/1.73 m^2^) or significant hepatic dysfunction (alanine transaminase >3* the upper limit of normal)^10, 11^, (2) patients with infections or autoimmune diseases and (3) patients with a history of malignancy. Written informed consent was received from all patients, and the study was passed by the ethics committee of the hospital and in accordance with the relevant guidelines and regulations.

### Clinical examination

SA levels and other blood variables were measured under fasting conditions in the morning of day following admission. SA was measured using a Beckman Coulter AU5821 or AU5831 (Beckman Coulter Inc, California). Echocardiography was routinely performed within 24 hours after admission to the hospital. Left ventricular ejection fraction (LVEF) was evaluated using the Simpson’s biplane method. The eGFR was calculated by the Modification of Diet in Renal Disease equation for Chinese patients^12^. Demographic and clinical characteristics including variables of Euro score were collected by one researcher with the use of an electronic case report form and then confirmed by another researcher. Euro score developed by Nashef SA *et al*. was calculated in this analysis^2^.

### Definition and end points

Hypoalbuminemia was defined as SA < 35 g/L. Stenosis of greater than 50% in the major coronary arteries was considered as coronary artery disease. The primary endpoint in this analysis was in-hospital mortality. All causes of death in the year after the operation, and in-hospital events (including death, dialysis and stroke) were considered as secondary end points.

### Statistical analysis

All statistical analyses were performed using SPSS software version 13.0 (SPSS, Inc., Chicago, Illinois). Patients were classified into two groups according to the SA level on admission: a non-hypoalbuminemic group and a hypoalbuminemic group. Continuous variables were shown as mean ± standard deviation or as medians and interquartile ranges, according to different distribution types. An independent sample T-test or non-parameters test was then performed, accordingly. Categorical variables were presented as absolute counts and percentages, and analyzed using the x2 test. Variables whose p value was less than 0.05 (except for components of the Euro score) in univariate logistic regression analysis were included in the multivariable analysis. In addition, these might affect SA level or prognosis in the clinical practice were also included in multiple analysis to rule out the interference. Receiver operating characteristic (ROC) curve analysis was conducted to determine the best cut-off value of SA and of the Euro score for predicting in-hospital mortality. The areas under the curve (AUCs) were compared using MedCalc statistical software (version 12.7.10, Ostend, Belgium). Continuous net reclassification improvement (NRI) was also used to discover the difference of predictive value. The Kaplan–Meier method was applied to assess the cumulative rate of 1-year mortality after the operation, and survival differences between groups were compared using the log-rank test. A 2-tailed P-value <0.05 was considered statistically significant.

## Results

### Baseline clinical characteristics

Between March 2009 and July 2013, 1858 patients with rheumatic heart disease undergoing valve replacement surgery and preoperative coronary angiography were screened. A total of two patients killed themselves during hospitalization and 40 patients did not have their SA levels measured upon admission. In addition, patients with eGFR <30 mL/min/1.73 m^2^ (n = 13) and alanine transaminase >120 U/L (n = 21) were excluded to avoid the confounding influence on albumin levels. Thus, 581 (32.6%) males and 1201 (67.4%) females were selected in this study with a mean age of 58 ± 6 years.

**Figure 1:**
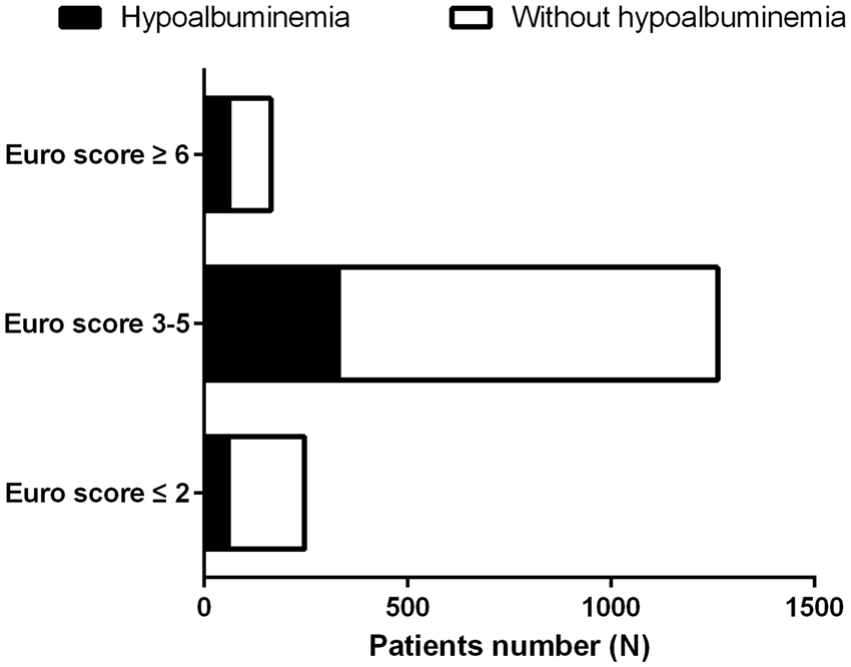
Distribution of patients with hypoalbuminemia with different Euro scores.

A total of 27.3% of patients also had hypoalbuminemia (SA < 35 g/L). In addition, there were 23.9% of patients with hypoalbuminemia in patients with a Euro score of ≤2, 26.1% in patients with a Euro score of 3–5 and 37.2% in patients with a Euro score of ≥6 (Fig. 1). Included patients were classified into two groups according to SA levels at admission: a non-hypoalbuminemic group (n = 1295) and a hypoalbuminemic group (n = 487). Patients with hypoalbuminemia were significantly older (58.2 ± 5.9 vs 57.4 ± 5.6, p = 0.005) and less likely to be female (62.8% vs 69.1%, p = 0.012). There was a larger proportion of smokers, New York Heart Association (NYHA) Functional Classification >II, pulmonary artery systolic pressure (PASP) > 60 mmHg and history of hypertension in the hypoalbuminemic group. Lower eGFR and LVEF were seen in patients with hypoalbuminemia in whom the Euro score was higher. A total of 72 patients died while in hospital: 40 (3.1%) in the non-hypoalbuminemic group and 32 (6.6%) in the hypoalbuminemic group. There was a statistically significant difference between the two groups (p = 0.001, Table 1).

Correlation analysis revealed that there was a negative relation between SA and C-reactive protein (CRP) (r = −0.212, p < 0.001).

### Role of hypoalbuminemia for in-hospital mortality

The univariate analyses for in-hospital mortality are shown in Table 2. Results revealed that age, previous valve replacement, NYHA Functional Classification > II, eGFR < 60 mL/min/1.73 m^2^, hypoalbuminemia, coronary artery bypass grafting (CABG) and Euro score were related to an increased in-hospital mortality. These variables (except for components of the Euro score) were studied by multiple logistic regression analysis, and it was revealed that hypoalbuminemia was a reliable predictor of in-hospital death (OR = 1.89, 95% CI, 1.14–3.13, P = 0.014), even after adjusting for the Euro score.

**Table 1:** Demographic and clinical characteristics in the two different preoperative plasma albumin groups.

Clinical variables	Non-hypoalbuminemic group (n = 1295)	Hypoalbuminemic group (n = 487)	P
Age (year)	57.4 ± 5.6	58.2 ± 5.9	*0*.*005*
Females, n (%)	895 (69.1)	306 (62.8)	*0*.*012*
Previous stroke, n (%)	46 (3.6)	13 (2.7)	0.353
Hypertension, n (%)	143 (11.0)	74 (15.2)	0.017
Diabetes mellitus, n (%)	76 (5.9)	34 (7.0)	0.384
Previous valve replacement	26 (2.0)	9 (1.8)	0.829
Coronary artery disease, n (%)	71 (5.5)	32 (6.6)	0.380
NYHA > II, n (%)	527 (40.7)	261 (53.6)	<*0*.*001*
Serum creatinine (µmol/L)	78.2 ± 20.5	85.3 ± 23.4	<*0*.*001*
eGFR (mL/min/1.73 m^2^)	90.4 ± 25.0	82.6 ± 23.0	<*0*.*001*
ALT (U/L)	21.0 (17.0, 27.0)	21.0 (16.0, 30.0)	*0*.*135*
SA (g/L)	38.9 ± 2.7	32.2 ± 2.6	<*0*.*001*
LVEF (%)	62.3 ± 8.6	59.8 ± 10.0	<*0*.*001*
Valve intervention			
Aortic	526 (40.6)	199 (40.9)	*0*.*925*
Mitral	1217 (94.0)	463 (95.1)	*0*.*375*
Tricuspid	990 (76.4)	401 (82.3)	*0*.*007*
CABG	63 (4.9)	21 (4.3)	*0*.*624*
Euro score	3.6 ± 1.3	3.9 ± 1.4	<*0*.*001*
In-hospital Death	40 (3.1)	32 (6.6)	*0*.*001*
In-hospital events	55 (4.2)	41 (8.4)	*0*.*001*

NYHA, New York Heart Association; eGFR, estimated glomerular filtration rate; ALT, alanine transaminase; SA, serum albumin; LVEF, left ventricular ejection fraction; CABG, coronary artery bypass grafting.

**Table 2:** Univariate analysis and multiple logistic regression analysis for in-hospital deaths.

Clinical variables	Univariate analysis		Multiple logistic regression		
	OR	P	OR	95% CI	P
Age	1.10	<0.001			
Female	0.75	0.247			
Hypertension	1.47	0.237			
Diabetes mellitus	1.97	0.081	1.79	0.81, 3.93	0.148
Previous valve replacement	4.18	0.004			
Coronary artery disease	1.51	0.347			
NYHA > II	2.17	0.002			
eGFR < 60 mL/min/1.73 m^2^	2.07	0.023			
ALT	1.01	0.267	1.01	1.00, 1.03	0.175
Hypoalbuminemia	2.21	0.001	1.89	1.14, 3.13	0.014
LVEF	0.98	0.104			
Aortic valve intervention	1.40	0.164			
Mitral valve intervention	0.55	0.142			
Tricuspid valve intervention	1.78	0.096			
CABG	2.69	0.012	2.50	1.11, 5.61	0.026
Euro score	1.62	<0.001	1.61	1.37, 1.89	<0.001

The variables in the Euro score were not included in the multiple analysis. NYHA, New York Heart Association; eGFR, estimated glomerular filtration rate; ALT, alanine transaminase; SA, serum albumin; LVEF, left ventricular ejection fraction; CABG, coronary artery bypass grafting.

**Figure 2:**
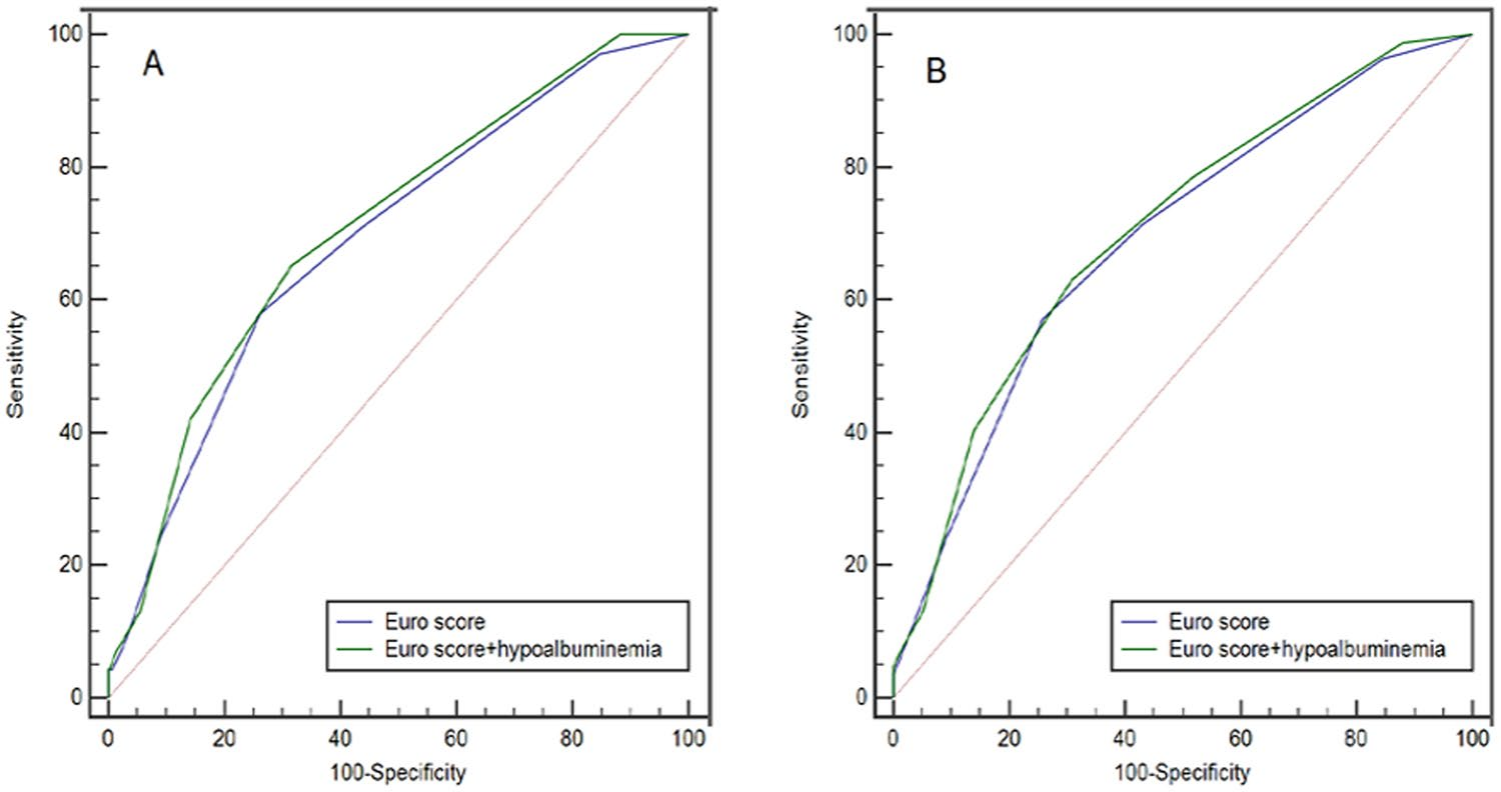
Comparison of areas under the curve for in-hospital (**A**) and 1-year mortality (**B**).

According to the odds ratio from multivariate analyses, we assigned one point for hypoalbuminemia. The associations between in-hospital mortality and the old and new risk stratification assessment score are displayed in Table 3. The new risk score was able to identify more low-risk patients than the old one. In addition, the ROC curve showed that SA < 36.6 had a sensitivity of 57.3% and a specificity of 63.9% for predicting in-hospital deaths (AUC = 0.623, 95% CI, 0.558–0.688, P < 0.001). For the Euro score, the AUC was 0.690 (95% CI 0.627–0.753, P < 0.001) and the sensitivity and specificity were 58.0% and 73.7%, respectively. There was no significant difference between their AUCs (z = 1.468, p = 0.142). The inclusion of hypoalbuminemia in the Euro score produced a slight improvement (z = 1.569, p = 0.117, Fig. 2). We also calculated the continuous NRI and the result demonstrated that the addition of hypoalbuminemia to Euro score enhanced net reclassification improvement for in-hospital mortality (NRI = 0.346, 95% CI: 0.108–0.584, P = 0.004).

**Table 3:** Risk of in-hospital death stratified by different Euro scores.

	Euro score		Euro score + hypoalbuminemia	
	N (%)	In-hospital mortality, %	N (%)	In-hospital mortality, %
Low, ≤2	247 (13.9)	2 (0.8)	188 (10.5)	0
Middle, 3–5	1262 (70.8)	50 (4.0)	1228 (68.9)	40 (3.3)
High, ≥6	164 (9.2)	17 (10.4)	257 (14.4)	29 (11.3)

### Hypoalbuminemia and 1-year mortality

**Figure 3:**
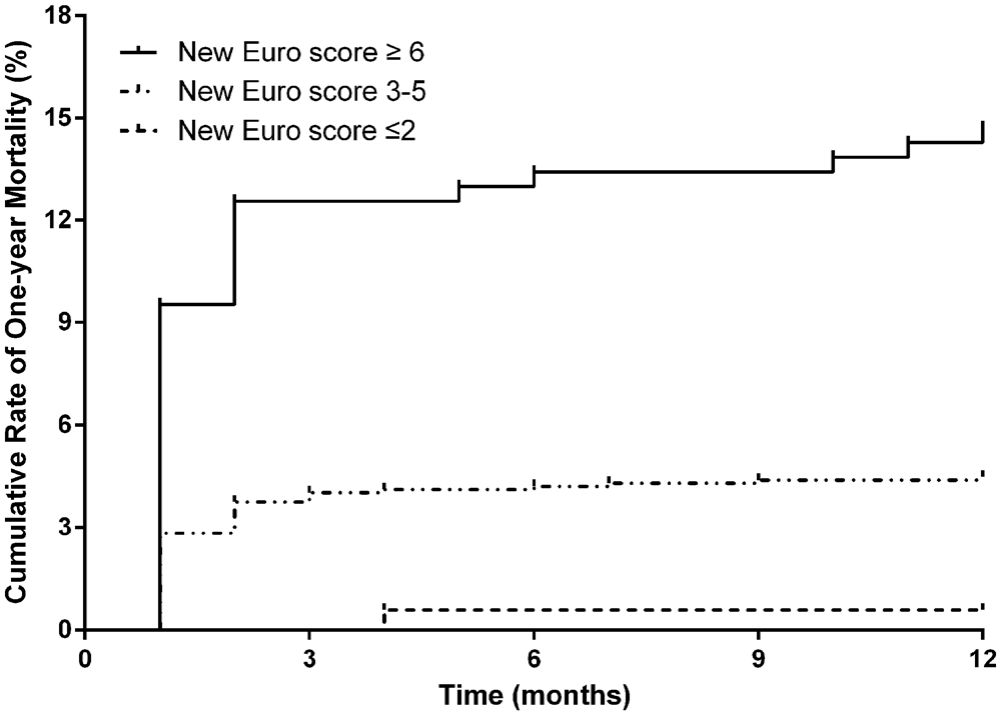
Cumulative rate of 1-year mortality after the operation, in patients with different risk scores.

All enrolled patients were followed up for 1 year after the operation. In this follow-up period, 193 (10.8%) patients were lost to follow-up and 87 (4.9%) died. The ROC curves of Euro score and new one showed no differences in their AUCs (z = 1.326, P = 0.185, Fig. 2). However, compared with the Euro score alone, combined use of hypoalbuminemia and Euro score was associated with significant improvements in the ability to predict 1-year death (NRI = 0.306, 95% CI: 0.091–0.521, P = 0.005). Kaplan-Meier curve analysis was shown in Fig. 3, revealing that the cumulative rate of 1-year mortality after the operation was higher in patients with a new Euro score of ≥6 (Log-rank = 47.72, p < 0.001). Multivariate Cox proportional hazard modelling revealed that new Euro score remained a significant predictor for 1-year death (HR = 1.56, 95% CI, 1.38–1.77, P < 0.001), even after adjusting potential risk factors (Table 4).

**Table 4:** Multivariate Cox proportional hazard modelling results of 1-year mortality.

Clinical variables	Hazard ratio (HR)	95% Confidence interval (CI)	p-value
Diabetes mellitus	1.55	0.78, 3.10	0.215
ALT	1.01	0.99, 1.02	0.427
CABG	2.41	1.24, 4.68	0.009
New Euro score	1.56	1.38, 1.77	<0.001

The variables in the New Euro score were not included in the multiple analysis. ALT, alanine transaminase; CABG, coronary artery bypass grafting.

## Discussion

This is the first study to assess the prognostic value in patients with RHD undergoing VRS. The main finding was that hypoalbuminemia was an independent risk factor for in-hospital and 1-year mortality after VRS in patients with RHD. In addition, including hypoalbuminemia in the Euro score produced a slight improvement, which might identify more patients at high risk of poor outcomes than the Euro score alone.

A total of 27.3% patients with RHD suffered from hypoalbuminemia on admission to this study, for three possible reasons. The first reason was the relationship between age and SA; Weaving *et al*. discovered that, at around age 20, SA reached a peak value and then decreased with age^13^. The average age in this study was 58 ± 6 years. Secondly, all the RHD patients in this study required an operation, which reflected the fact that patients experienced symptoms such as fatigue, loss of appetite and shortness of breath, which could influence the protein intake. Thirdly, patients with RHD often suffer from cardiac dysfunction^14^. Previous investigations have indicated that hypoalbuminemia was common in patients with stable chronic^15^ or acute heart failure^16^. Hepatocellular function, nutritional status, inflammation and congestion may account for this phenomenon^17^.

This study demonstrated that preoperative hypoalbuminemia was related to poor outcomes in patients with RHD undergoing VRS. This could be explained by several theories. SA is a good index for indicating nutritional status^18^, and malnutrition has been reported to be associated with increased mortality in cardiac surgery patients^19^. Low SA, as a sign of malnutrition, was shown to be an independent predictor of increased mortality after cardiac surgery^20^. SA has also been associated with inflammation, in which the catabolism will increase, including reduced protein synthesis rates and increased protein degradation rates^21^. Therefore, hypoalbuminemia could represent inflammatory conditions. In this study, we also found that SA was negatively correlated with CRP. In addition, SA participated in modulating prostaglandin E2 (PGE2)-mediated immunosuppression^22^. Hypoalbuminemia has been shown to be a risk factor for surgical site infection after a gastrointestinal operation^23^. Another theory that can be considered is the relationship between low SA and postoperative acute kidney injury (AKI). Lee *et al*. reported that hypoalbuminemia was an independent predictor for AKI and that AKI was related to poor outcomes after off-pump coronary artery bypass surgery^24^. In the meta-analysis conducted by Wiedermann, a similar result was shown in that low SA was associated with AKI and mortality following development of AKI^25^. All these results support our data.

The mortality rate in this analysis was 4.0%, which was in accordance with a previous study^26^. Euro score is a risk assessment method that can calculate operative mortality in patients after cardiac surgery. However, the Euro score could not identify all patients at risk of death^27^. In this study, we found that adding hypoalbuminemia to the Euro score might identify more patients at high risk of poor outcomes than the Euro score alone. In clinical practice, patients presenting with higher Euro score were at high risk of poor outcomes and may need more attention and care in cases with hypoalbuminemia and high Euro score.

### Limitations

Several limitations should be considered in this analysis. Firstly, this study was a retrospective analysis based on prospectively collected data. Therefore, some admission SA levels were missing or confounding, which may affect the results. Secondly, SA was not dynamically detected. The relationship between prognosis and SA measured at different time points is unknown. Future studies should be performed to confirm whether receiving albumin treatment could improve outcomes.

## Conclusions

This study demonstrated that hypoalbuminemia was an independent risk factor for in-hospital and 1-year mortality after VRS in patients with RHD. In addition, inclusion of hypoalbuminemia in the Euro score produced a slight improvement in prognosis, which might identify more patients at high risk of poor outcomes than the Euro score alone.
